# The C-Terminal Half of SARS-CoV-2 Nucleocapsid Protein, Industrially Produced in Plants, Is Valid as Antigen in COVID-19 Serological Tests

**DOI:** 10.3389/fpls.2021.699665

**Published:** 2021-07-27

**Authors:** Laura Williams, Silvia Jurado, Francisco Llorente, Alberto Romualdo, Sara González, Ana Saconne, Isabel Bronchalo, Mercedes Martínez-Cortes, Beatriz Pérez-Gómez, Fernando Ponz, Miguel Ángel Jiménez-Clavero, Pablo Lunello

**Affiliations:** ^1^Agrenvec S.L., Madrid, Spain; ^2^Centro de Investigación en Sanidad Animal (CISA), Centro Nacional Instituto de Investigación y Tecnología Agraria y Alimentaria (INIA-CSIC), Valdeolmos, Spain; ^3^Madrid Salud: Public Health Services of Madrid City Council, Madrid, Spain; ^4^National Centre for Epidemiology, Instituto de Salud Carlos III, Madrid, Spain; ^5^Consortium for Biomedical Research in Epidemiology and Public Health (CIBERESP), Madrid, Spain; ^6^Centro de Biotecnología y Genómica de Plantas, Universidad Politécnica de Madrid-Instituto Nacional de Investigación y Tecnología Agraria y Alimentaria (CBGP, UPM-INIA, CSIC), Madrid, Spain

**Keywords:** COVID-19, SARS CoV-2 nucleocapsid protein, biofactories, plant expression, molecular farming, ELISA coronavirus

## Abstract

**Background:**

The fight against the current coronavirus disease 2019 (COVID-19) pandemic has created a huge demand of biotechnological, pharmaceutical, research and sanitary materials at unprecedented scales. One of the most urgent demands affects the diagnostic tests. The growing need for rapid and accurate laboratory diagnostic tests requires the development of biotechnological processes aimed at producing reagents able to cope with this demand in a scalable, cost-effective manner, with rapid turnaround times. This is particularly applicable to the antigens employed in serological tests. Recombinant protein expression using plants as biofactories is particularly suitable for mass production of protein antigens useful in serological diagnosis, with a neat advantage in economic terms.

**Methods:**

We expressed a large portion of the nucleoprotein (N) derived from SARS-CoV-2 in *Nicotiana benthamiana* plants. After purification, the recombinant N protein obtained was used to develop an indirect enzyme-linked immunosorbent assay (ELISA) for detection of antibodies to SARS-CoV-2 in human sera. To validate the ELISA, a panel of 416 sera from exposed personnel at essential services in Madrid City Council were tested, and the results compared to those obtained by another ELISA, already validated, used as reference. Furthermore, a subset of samples for which RT-PCR results were available were used to confirm sensitivity and specificity of the test.

**Results:**

The performance of the N protein expressed in plants as antigen in serologic test for SARS-CoV-2 antibody detection was shown to be highly satisfactory, with calculated diagnostic sensitivity of 96.41% (95% CI: 93.05–98.44) and diagnostic specificity of 96.37 (95% CI: 93.05–98.44) as compared to the reference ELISA, with a kappa (K) value of 0.928 (95% CI:0.892–0.964). Furthermore, the ELISA developed with plant-derived N antigen detected SARS-CoV-2 antibodies in 84 out of 93 sera from individuals showing RT-PCR positive results (86/93 for the reference ELISA).

**Conclusion:**

This study demonstrates that the N protein part derived from SARS-CoV-2 expressed in plants performs as a perfectly valid antigen for use in COVID-19 diagnosis. Furthermore, our results support the use of this plant platform for expression of recombinant proteins as reagents for COVID-19 diagnosis. This platform stands out as a convenient and advantageous production system, fit-for-purpose to cope with the current demand of this type of biologicals in a cost-effective manner, making diagnostic kits more affordable.

## Introduction

The present SARS-CoV-2 coronavirus pandemic is the most important global sanitary crisis humankind has faced in the last 100 years. Since its emergence in Asia at the end of 2019, the new virus has already hit all continents, producing harm of remarkable magnitude both in public health, and in the World economy. The pandemic has also underscored the need of rapid and flexible responses in the industrial capacities required to produce large amounts of tools and reagents for diagnosis, therapies, and prevention (vaccines) of new viral diseases in a cost-effective way.

Serological tests are essential tools for the detection of antibody responses to SARS-CoV-2 infection. They play an essential role not only in diagnosis but also in the evaluation of the seroprevalence in exposed populations and in research studies aimed to provide answers to still open key questions regarding the complete characterization of the course of the infection such as, for instance, the duration of antibodies. In this regard, enzyme-linked immunosorbent assays (ELISAs) are widely used in most diagnostic and research applications due to their long-proved efficiency, easiness, and high throughput capacity.

Many serological assays have been made available recently for detection of SARS-CoV-2 specific antibodies (Hoste et al., 2020; Rikhtegaran Tehrani et al., 2020; Whitman et al., 2020). However, their relatively high cost still prevents many laboratories, especially in developing countries, from their broad use. Therefore, approaches enabling significant reductions in production costs for these assays are highly desirable. Specifically, as the most expensive component of these assays, the production of the recombinant viral proteins used as antigens would benefit largely of more cost-efficient processes, leading to more affordable serological tests.

A particularly attractive system for cost-efficient recombinant protein production is based on their expression in plants, often named Plant Molecular Farming, which has gained importance and relevance over the years. Nowadays, several proteins with pharmaceutical and other industrial applications are already being produced in plants, particularly in *Nicotiana benthamiana*. The favorable features of this industrial approach have been widely and frequently reviewed, among which speed of production in very large amounts and comparative low costs of productions stand out (Ma et al., 2003; Williams et al., 2014, 2016; Moon et al., 2019; Capell et al., 2020; McDonald and Holtz, 2020). In the particular case of SARS-CoV-2 proteins, a few examples showing their production in plants have been published very recently (Diego-Martin et al., 2020; Siriwattananon et al., 2021). A previous approach to produce plant-made viral proteins that could be potentially used as antigens has been shown for the related SARS-CoV, where antibody binding to immobilized plant-produced virus antigen was demonstrated using a limited panel of serum samples (Demurtas et al., 2016).

Many serological assays for SARS-CoV-2 antibody detection rely upon the use of the viral multifunctional nucleoprotein (N) (McBride et al., 2014; Hoste et al., 2020; Tré-Hardy et al., 2021), expressed in recombinant forms using different expression systems. We have undertaken the production of a large portion of SARS-CoV-2 N protein in *N. benthamiana*, through its transient expression from a vector derived from *Tobacco mosaic virus* (TMV) and shown its suitability to detect antibodies using a wide panel of human serum samples obtained in early 2020. For that, we developed an ELISA test which was used to analyze the presence of antibodies to SARS-CoV2 N antigen in the panel sera. The results were compared with those obtained using an already validated ELISA test commercially available.

## Materials and Methods

### Cloning of SARS-CoV-2 N Gene Into Plant Expression Vector TMV-PSN

#### Generation of Infectious RNA

**FIGURE 1:**
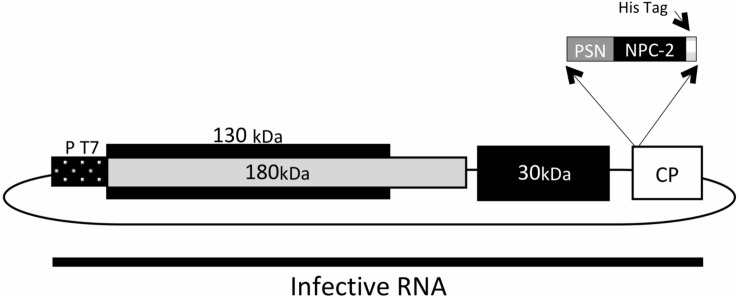
Schematic diagram of the expression vector TMV-PSN-NPC-2 DNA. The coding sequence of SARSCoV-2 Nucleoprotein (NPC-2) was modified by the addition of C-terminal His tag and inserted into the TMV vector. An 82 bp fragment corresponding to the signal peptide (PSN) was included in position N-terminal. Infectious cDNA clone was transcribed from the T7 bacteriophage RNA promoter (PT7), and the RNA used to infect *N. benthamiana* plants. The TMV genome has four open reading frames (ORF). ORF 1 of 130 kDa with Methyltransferase and Helicase function, ORF 2 of 180 kDa with Methyltransferase, Helicase function and RNA-dependent RNA polymerase (RdRp) function. ORF 3 of 30 kDa corresponding to Movement Protein (MP) and ORF 4 of 17 kDa corresponding to Coat Protein (CP).

The cDNA encoding for a truncated N form (termed “NucleoProtein Coronavirus-2,” abbreviated, NPC-2) of SARSCoV-2 which codes for its amino acids 231–419 (GenBank YP_009724397.2) was designed with a His-tag at its C-terminus. The synthetic sequence was made by GENEART AG optimizing for codon usage in *N. benthamiana* plants. The sequence was cloned into TMV expression vector (GENEWARE System) under the control of a duplicated promoter of the capsid protein (CP) gene. The extensin signal peptide (PSN) from *Nicotiana tabacum*, which directs the recombinant NPC-2 toward the plant endoplasmic reticulum (Figure 1), was also included.

Plasmid DNA of the stable clone TMV-PSN-NPC-2 was purified using QIAGEN’s plasmid purification kit, according to the manufacturer’s instructions.

**FIGURE 2:**
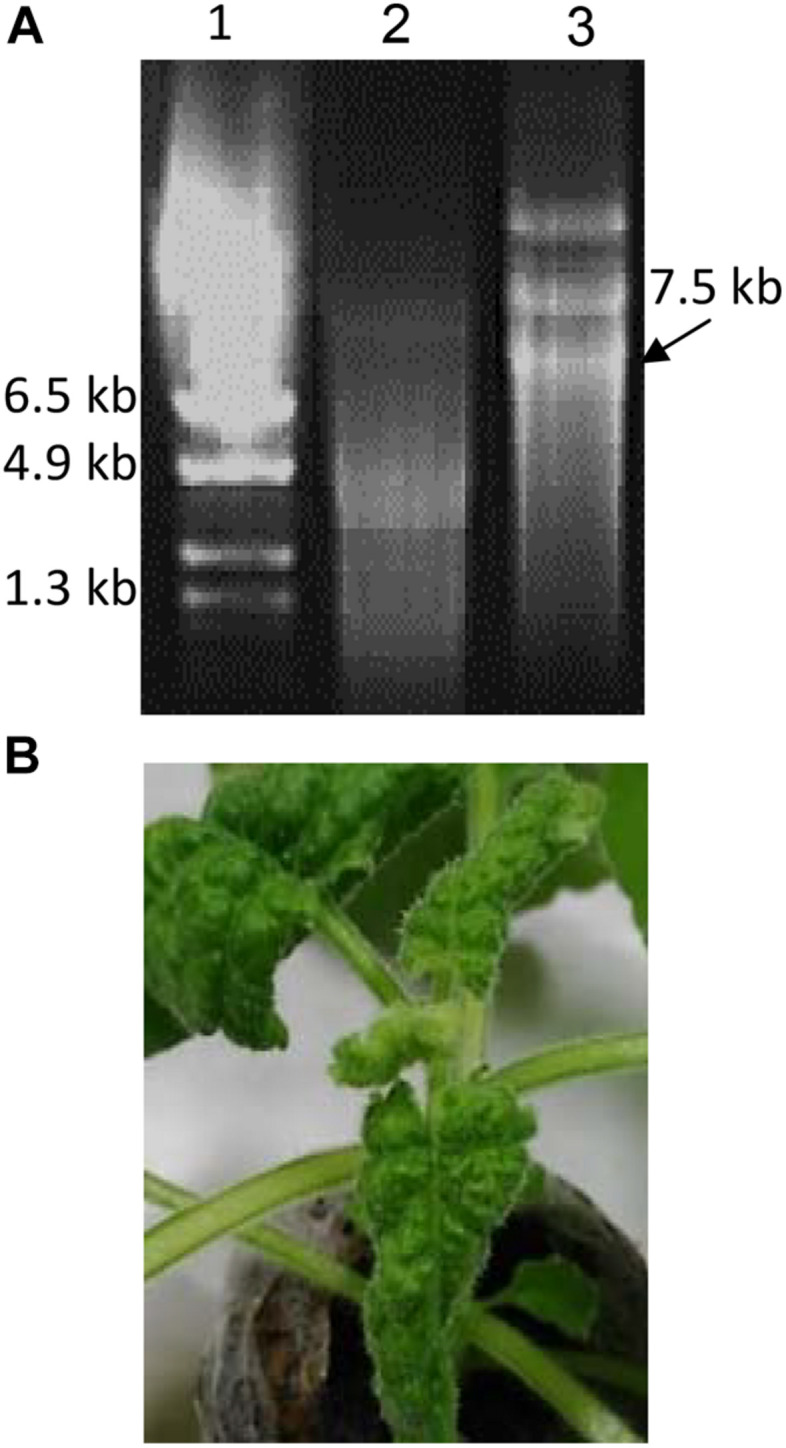
Production of infectious RNA from TMV-PSN-NPC2 clone and inoculation of *N. benthamiana* plants **(A)** *In vitro* transcription (IVT), ethidium bromide-stained 0.5% agarose gel. 1: RNA marker (Promega G319A), 2: RNA control kit, 3: RNA TMVPSN-NPC2. The RNA transcribed (7.5 kb) was used for plant inoculation. **(B)** Infectivity of the recombinant virus. The picture shows a plant at 8 dpi (days post inoculation). Leaves with symptoms of infection reflected by the mild leaf deformation with variable mottling grade.

Capped infectious RNA was prepared *in vitro* from plasmid TMV-PSN NPC-2 clone with the mMESSAGE mMACHINE^TM^ T7 Transcription Kit (INVITROGEN). Each *in vitro* transcription reaction (IVT) was made with 0.625 μl plasmid DNA (0.2 μg/μl), 0.125 μl water, 0.25 μl 10× Buffer, 1.25 μl 10× nucleotides (CAP) and 0.25 μl 10× T7 RNA polymerase, in a 2.5 μl final volume. The reaction was incubated for 2 h at 37°C. RNA was quantified by electrophoresis in a 0.5% ethidium bromide-stained agarose gel (Figure 2A).

#### Inoculation of *Nicotiana benthamiana* Plants With the Infectious Transcript

Three hundred *N. benthamiana* plants, 18–28 day-old, were inoculated by gently scraping their leaves with 50 μl of diluted infectious RNA [2.5 μl of IVT reaction in 100 μl of 1× FES buffer (43 mM glycine, 139 mM K_2_HPO4, 1% sodium pyrophosphate, 1% bentonite and 1% of celite, w/v)]. Plants were kept at 25°C in growth chambers (60% humidity, 16/8 photoperiod).

Infected leaves from 5 dpi symptomatic plants were pooled and extracted by grinding in 150 ml of 50 mM phosphate buffer, pH 7.5, with 0.04% (w/v) of sodium metabisulphite. The crude extract was then used to inoculate 2,000 *N. benthamiana* plants in a greenhouse (70% humidity, 16/8 photoperiod). Infection and NPC-2 expression were assessed by ELISA with polyclonal antibodies against TMV (AGDIA, CAB 57400) and SARSCoV-2 N protein (Invitrogen, PA5-81794) according to the manufacturers’ instructions.

Stability of the recombinant virus during infection was evaluated by Immunocapture Reverse Transcription Polymerase Chain Reaction (IC-RT-PCR) according to Nolasco et al. (1993). To amplify the NPC-2 insert from viral vector, two specific oligonucleotides were designed hybridizing outside of the vector cloning site (cTMVF 50 -CGT GTG ATT ACG GAC ACA ATC C-30 and cTMVR 50 -TAC TGT CGC CGA ATC GGA TTC G-30). Thereby, the expected size of the complete construction (TMV–PSN–NPC-2) is 820 bp and for the “empty” vector (TMV–PSN) 226 bp. The infected plants with viral RNA were used to infect plants by mechanical inoculation. The stability of the recombinant viral vector was evaluated in this way in plants at 8- and 12-days post inoculation (dpi).

#### Purification and Quantification of Recombinant NPC-2

Symptomatic leaves were harvested 11–13 dpi and blender-ground in 100 mM potassium phosphate buffer, pH 7.3–7.6, 100 mM NaCl, 0.04% (v/v) Triton ×100, 5 mM BME, and 0.1 mM PMSF (1:3 w/v). The homogenate was filtered through three cloth layers and centrifuged at 6,000 × *g* to remove cell debris and insoluble material. The soluble extract was subjected to a two-step purification process involving a first stage by Ni^2+^-sepharose affinity chromatography, and a second stage by Cation Exchange in a HiTrap SP Sepharose FF 5 ml (AKTA Xpress System -GE), according to Williams et al. (2016). The absorbance (280 nm wavelength) peaks were analyzed on a 15% SDS-PAGE and by western blot (as described below). Purified NPC-2 protein was quantified by SDS-PAGE and UV spectrophotometry by Nanodrop.

#### Molecular and Antigenic Characterization of NPC-2

The purified recombinant NPC-2 protein was subjected to SDS-PAGE gel analysis (100–500 ng/lane) and stained with Coomassie Blue. The bands with a migration in the gel around 21 kDa, were excised. Their tryptic fragments were analyzed by MALDI-TOF in an external service (SCSIE-Universidad de Valencia).

The purified NPC-2 was subjected to Western blot analysis, probed with a commercial polyclonal antibody specific for SARSCoV-2 N protein (N-full length SARSCoV-2 Nucleocapsid Polyclonal Antibody, Invitrogen PAS81794). For that, recombinant NPC-2 protein (0.3–3.0 μg) was separated by SDS-PAGE and electrotransferred into a nitrocellulose membrane, which was blocked with TBS buffer supplemented with 5% (w/v) skimmed milk, and subsequently incubated with the commercial anti-SARSCoV-2 N polyclonal antibody, diluted 1:2,000, for 1 h. The development was done after incubation with alkaline-phosphatase-conjugated goat anti-rabbit IgG (97048 Abcam), 1:200, 1 h, followed by substrate addition (NBT/BCIP).

### Serological Validation of Plant NPC-2 With Samples From SARS-CoV2-Infected and Non-infected Individuals

#### Serum Samples

In this study we used a total of 416 human serum samples from the “Program of Surveillance and Early Detection of COVID 19 in essential services personnel in the city of Madrid” collected in April 2020, when the city of Madrid was severely hit by the pandemic, which were provided by the Institute of Public Health of the Madrid City Council. Samples were classified as positive (*n* = 193) or negative (*n* = 223) to SARS-CoV-2 antibodies on the basis of their result with a commercially available ELISA (INgezim COVID 19 DR, Ingenasa). already validated, with 100% diagnostic sensitivity (95% confidence interval, CI = 97.7–100%) and 98.2% diagnostic specificity (95% CI = 97–99.1%) (Hoste et al., 2020). A subset of the samples corresponded to patients with confirmed SARS-CoV-2 infection by a positive result by Real Time Reverse Transcription Polymerase Chain Reaction RRT-PCR (Corman et al., 2020) in oropharyngeal samples at the time of serum collection, or previously (Table 1).

**TABLE 1:** Serum classification by commercial serological assay and PCR confirmation.

	Serum samples
Antibody + /confirmed by PCR	86
Antibody + /not confirmed by PCR	107
Antibody -/confirmed by PCR	7
Antibody -/not confirmed by PCR	216
Total	416

#### Indirect NPC-2 ELISA

Enzyme-linked immunosorbent assay plates (Maxisorp, NUNC, Roskilde, Denmark) were coated with 2 μg/well of purified recombinant NPC-2 protein antigen in 0.1 M carbonate/bicarbonate buffer pH 9.6 and maintained at 37°C for 45 min. Thereafter, wells were blocked with phosphate buffered saline, pH 7.4, 0.05% (v/v) Tween 20 (PBST), containing 3% (p/v) skimmed milk, for 1 h at room temperature (RT). The plates were incubated with serum samples diluted 1:200 in PBST with 1% skimmed milk, for 2 h at RT. Duplicates of positive (sera from a confirmed COVID 19 patient), and negative controls were included in each plate. Plates were washed three times with PBST and incubated with horseradish peroxidase (HRP)-conjugated goat anti-human IgG (Thermofisher), diluted 1:5,000 in PBST, for 1 h at RT. Finally, after three additional washes with PBST, plates were incubated with peroxidase substrate (TMB-MAX, Neogen Corporation) for 5 min in the dark at RT. Reaction was stopped by adding 0.5 M H_2_SO_4_. Optical density (OD) was measured in each well at 450 nm in an ELISA microplate reader. Results were valid when OD in negative control wells was below 0.8 OD units and the OD in positive control wells was over 2.50 OD units.

#### Statistical Analysis

Data were statistically analyzed using a receiver-operator characteristics (ROC) curve analysis using the GraphPad Prism 6 software. The kappa statistic, analyzed by the same software, was used to measure the strength of agreement between the results obtained by the indirect ELISA test with recombinant NPC-2 protein antigen and the reference commercial ELISA.

Sensitivity of the tests (NPC-2 ELISA and reference ELISA) was calculated as the proportion of positive samples by the method used as reference (reference ELISA or samples from patients with previous positive PCR result) that tested also as positive in the evaluated test.

Specificity was calculated as the proportion of negative samples by reference ELISA which tested negative in NPC3 ELISA.

## Results

### Cloning of SARSCoV-2 N Gene Into Plant Expression Vector TMV-PSN

#### Production of Infectious RNA

The DNA sequence of SARSCoV-2 N (Gen Bank YP_009724397.2, aa 231–419), designed with His-tag C-terminal and optimized to achieve its correct expression in *N. benthamiana* plants was successfully cloned into TMV-PSN vector and the recombinant TMV-PSN-NCP2 clone was confirmed by sequencing. *In vitro* 6 kb RNA transcripts produced from the recombinant TMV-PSN-NCP2 clone were used to infect plants by mechanical inoculation (Figure 2A). Evident symptoms of infection such as leaf deformation with variable mottling grade were visible after 6–8 dpi (Figure 2B).

#### Stability of TMV-PSN NPC-2 in *Nicotiana benthamiana* Plants

**FIGURE 3:**
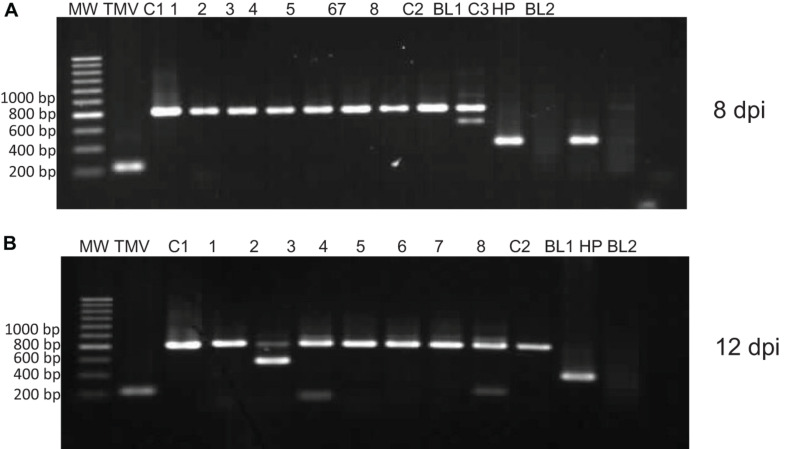
Stability of NPC-2 by IC-RT-PCR. Amplification of an 820-bp fragment, which is the size expected for the recombinant clone, was observed in 100% of the plants infected. MW, hiperladder 200 bp; TMV, empty vector, C1, TMV-PSN-NPC2 clone (820 pb). 1–8, Infected plant, C2, TMV-PSN-EGF clone (397 pb), BL1, negative PCR control; C3, infected plant with TMV-PSN-EGF (IC-RT-PCR positive control). HP: healthy plant, BL2: negative control of IC-RT-PCR. **(A)** Infected plants to 8 dpi and **(B)** infected plants to 12 dpi.

The stability of the TMV-PSN-NPC2 construct plasmid was analyzed in the inoculated *N. benthamiana* plants by IC-RT-PCR at 8 and 12 dpi. Amplification of an 820-bp fragment, which is the size expected for the recombinant clone, was obtained in 100% of the plants analyzed with TMV-PSN-NPC-2 while in the different controls included, namely, extracts from plants infected with the empty vector (TMV-PSN) and from plants infected with another unrelated construct (TMV-PSN EGF) the amplicons observed were of 226 and 397 bp, respectively, corresponding with their expected sizes (Figure 3).

The IC-RT-PCR assay not only showed that 100% plant leaves analyzed had the expected size to insert NPC-2 (820 bp) but also that 1 in 8 plants, both in 8 and 12 dpi, showed a partial deletion of the insert (600 bp).

#### Purification From *Nicotiana benthamiana* Leaves and Yield Evaluation

**FIGURE 4:**
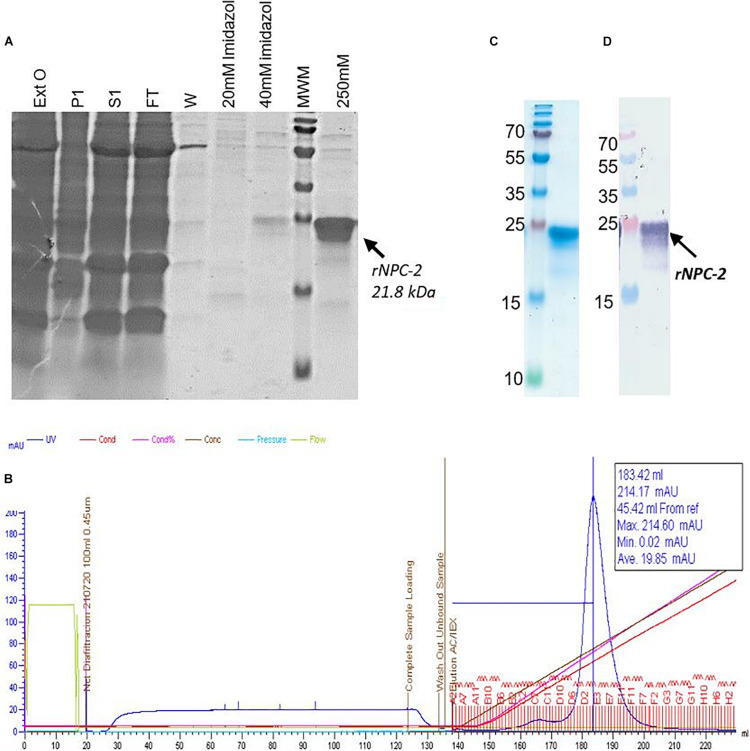
Analysis of purified plant-produced recombinant NPC-2. See main text for details of the purification procedure. **(A)** Coomassie stained SDS-PAGE of Affinity Chromatography (IMAC-resin Ni2 + Sepharose Fast Flow). Fifteen μl of: Ext-0 plant extract (Ext0), pellet (P1), soluble plant extract (S1), FT flowthrough (FT), washed (W) and imidazole gradient (20, 40, and 250 mM) were loaded. The NPC-2 protein was eluted with more than 80% of purity and migrate in SDS-PAGE (reducing conditions) as a protein of 21.8 kDa. **(B)** Fractions recovered from Cation Exchange Chromatography, (Hitrap SP FF SP) corresponding to recombinant NPC2. **(C)** Final Purity of recombinant NCP2 obtained from NaCl gradient analyzed by SDS-PAGE stained with Coomassie Blue. **(D)** Western blot analysis of recombinant protein NCP2 identified by Nucleocapsid Polyclonal Antibody (Invitrogen PAS81794).

The recombinant protein NPC-2, was purified from leaf extracts, using two successive chromatographic steps, Ni^2+^-affinity and cationic exchange. After Ni^2+^-affinity chromatography, more than 90% of the NPC-2 protein was recovered from the soluble plant extract, with over 80% purity (Figure 4A). NPC-2 migrated as a double band with an apparent molecular weight 21.8 kDa, in agreement with our predictions based on amino acid sequence.

After the second purification step, cation exchange column chromatography, one major protein peak was observed on a 0–1 M NaCl gradient (Figure 4B). The recombinant protein typically eluted at 30.2% of elution buffer (0.3 M NaCl approximately). Fractions were pooled, concentrated and quantified by spectrophotometry. Upon Western blot analysis probed with anti SARS CoV-2 N antibody, the 20–22 kDa bands were antigenically identified as N-derived NPC-2. We estimate a final NPC-2 yield of 200 mg/kg of fresh tissue, with over 95% homogeneity (Figures 4C,D).

MALDI-TOF analysis of the two bands recognized by the polyclonal SARS CoV-2 N antibody gave the same peptide mass finger printing pattern as the C-terminal (aa 231–419) of SARS CoV-2 N protein (data not shown).

### Serological Validation of Plant-Produced NPC-2 With Serum Samples From SARS-CoV2-Infected and –Non-infected Individuals

**FIGURE 5:**
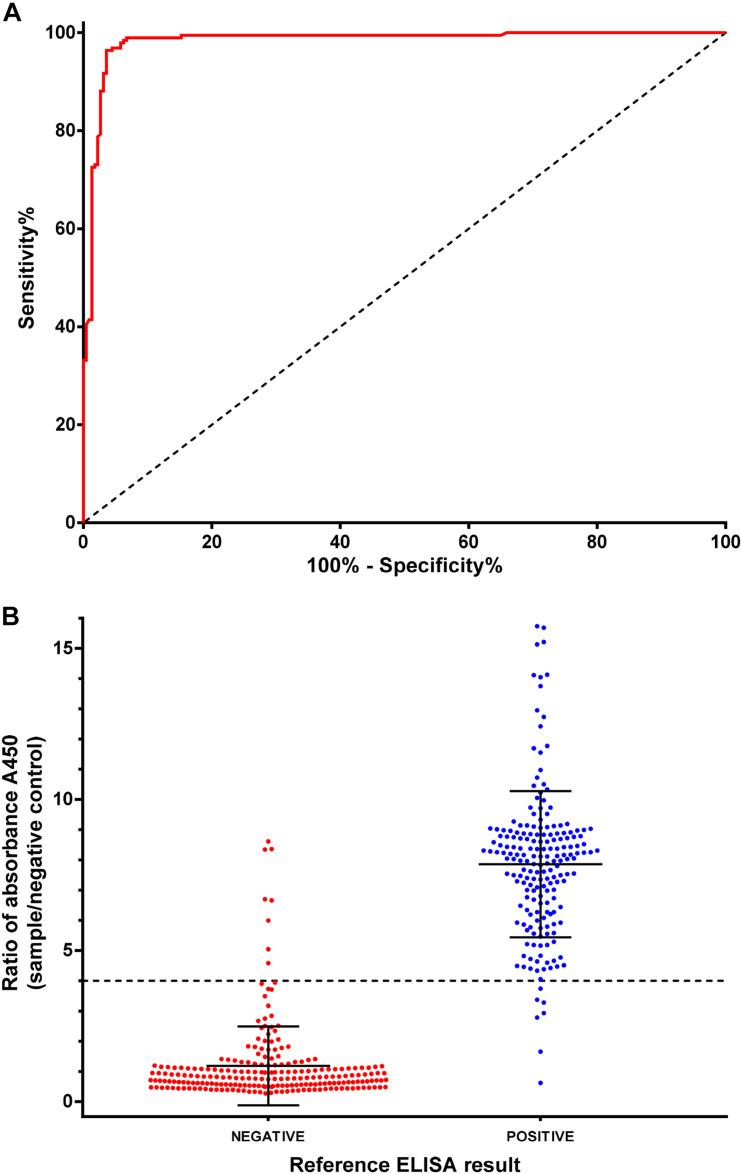
ELISA assay validation and diagnostic performance. **(A)** Receiver operating characteristic (ROC) analyses of the Indirect NPC-2 ELISA in 416 human sera, positive or negative by reference ELISA (INgezim COVID-19 DR, Ingenasa). **(B)** Distribution of NPC-2 ELISA protocol results by sample classification (negative or positive in reference ELISA test).

An indirect ELISA was developed based on the recombinant NPC-2 protein from *N. benthamiana* plants (hereinafter, NPC-2 ELISA). The ELISA was optimized for IgG antibody detection in serum samples (data not shown). A total of 416 human serum samples were analyzed, that, in the absence of a serological gold standard method, were previously classified as positive or negative on the basis of the results obtained with a commercial ELISA (hereinafter “reference ELISA”) that has proven to be highly sensitive and specific (Hoste et al., 2020). Importantly, the method chosen as reference is also based on the use of a recombinant N derived from SARS-CoV2. The results obtained with the NPC-2 ELISA (expressed as corrected OD values, i.e., O.D. ratios relative to negative serum control result) were compared with those from the reference ELISA. To compare the performance of the NPC-2 ELISA to that of the reference ELISA, a receiver-operator characteristics (ROC) analysis was used (Figure 5A). The area under the curve (AUC) of the NPC-2 ELISA was 0.9831 [95% confidence interval (CI): 0.971–0.995] indicating that this method has a very high diagnostic accuracy.

Based on the ROC curve, a cut-off of four was set (Figure 5B). Using this value, a diagnostic specificity of 96.41% (95% CI: 93.05–98.44) and sensitivity of 96.37 (95% CI: 93.05–98.44) were achieved (Figure 5B and Table 2). Using the kappa statistic to measure the concordance of results between techniques, a value of K = 0.928 was obtained, indicating an almost perfect agreement between reference and the NPC-2 ELISA (Landis and Koch, 1977).

**TABLE 2:** Diagnostic sensitivity (DSe), diagnostic specificity (DSp) and kappa coefficient (K) of the NPC-2 ELISA compared to the reference ELISA.

		NPC-2 ELISA		
		Positive	Negative	Total
Reference ELISA	Positive	186	7	193
	Negative	8	215	223
	Total	194	222	416

DSe	96.41% (95% CI: 93.05–98.44)			
DSp	96.37 (95% CI: 93.05–98.44)			
K value	0.928 (95% CI:0.892–0.964)			

As a step further in the validation of the NPC-2 ELISA, the results obtained in a subset of serum samples selected as “true positive” i.e., from individuals with a positive PCR result either in the same day of serum collection or in previous analyses, were analyzed. Table 3 shows a comparison of the results obtained in these true positive samples by NPC-2 ELISA and reference ELISA tests. NPC-2 ELISA detected 90.32% (84/93) of true positive sera, whereas the reference ELISA detected 92.47% (86/93), that is, the NPC-2 ELISA detected only a slightly lower proportion of true positive samples. In fact, only two samples with a positive result in the reference ELISA showed a negative result with NPC-2 ELISA, further confirming that both methods have a similar capacity to detect serologically positive COVID-19 cases.

**TABLE 3:** Comparison of ELISA assays in determining SARS-CoV-2 antibodies in serum samples from patients confirmed by RT-PCR.

		NPC-2 ELISA		
		Positive	Negative	Total
Reference ELISA	Positive	84	2	86
	Negative	0	7	7
	Total	84	9	93

## Discussion

The present paper reports a fast, efficient, and easily scalable expression system for the large-scale production of recombinant SARSCoV-2 nucleoprotein (N), useful as antigen for serologic tests, in plants of *N. benthamiana*. The availability of such production systems for viral proteins at a massive scale has become already a critical issue in the fight against COVID-19. Indeed, it is expected to become even more relevant in the years to come when epidemiological studies will need to be undertaken at a global level to conduct serological surveillance studies of the human population, as well as in animal reservoirs. The N protein of the virus is one of the several viral proteins potentially useful as antigenic markers of infection, together with S, E, and M, and probably the most widely used at this respect (Liu et al., 2020; Rikhtegaran Tehrani et al., 2020). Each one of these has pros and cons, so probably a global picture will be obtained through their combined use. Importantly, antibodies to N are not present in individuals vaccinated with S-based vaccines, so unlike S-based serologic tests, which effectively detect antibody responses elicited either by infections or by vaccines (mostly based on the development of immune responses to S protein, involved in seroneutralization and thus protection from SARS-CoV2 infections), N-based serologic test will be expected to react to antibodies raised by actual infections, and not vaccinations (as long as they are carried out using S-protein-based immunogens), which might be an advantage for some applications, e.g., in seroprevalence studies focused on infections, during ongoing or after vaccination programs. Some of the main characteristics of the N protein for these purposes have been recently reviewed (Li and Li, 2021).

The production of SARS-CoV-2 recombinant N protein has been addressed so far mostly in *Escherichia coli* (Algaissi et al., 2020). However, this prokaryote expression system frequently fails to fold correctly proteins that are to be acting in a complex eukaryotic environment. This incorrect folding usually results in the expression of insoluble conformations, forming inclusions, useless in most biotechnological applications. This problem has been found for the N protein. Plants are a good alternative for the production of eukaryotic proteins, particularly using transient expression systems based on viral vectors (Gleba et al., 2007; Pogue et al., 2010). In fact, some SARS-CoV-2 proteins have already been made in plants (Burnett and Burnett, 2019; Sainsbury, 2020; Makatsa et al., 2021; Siriwattananon et al., 2021), including N (Diego-Martin et al., 2020), although the suitability of plant-made N for detection and diagnosis of antibodies in samples of human sera has not been tested yet. In addition to these technical advantages, plant production of recombinant proteins presents an important economic advantage in terms of production costs, which becomes an important aspect when massive production is required. It has been estimated that these costs represent a mere 0.1% of mammalian cell-based platforms, or 2–20% of systems based on bacteria (Yao et al., 2015).

In this study, the C-terminal domain of the N protein (aa 231–419) has been expressed in *N. benthamiana* using the TMV-PSN vector, already used by our group for the expression of other proteins (Williams et al., 2016, 2014). The protein with a MW of 21.9 kDa was purified from soluble plant extract with a final yield of approximately 200 mg recombinant protein per kg of fresh tissue. This is a better yield in comparison with our previous results obtained by us for human vascular endothelial growth factor VEGF (Williams et al., 2016), and also a better expression level than the one obtained by others in plants for this protein (Diego-Martin et al., 2020). An initial number of 300 plants grown under controlled conditions (growth chambers) allowed us to generate enough inoculum to infect 11,000 plants in the greenhouse. This means processing 16 kg of plant tissue per week. With a yield of 200 mg per kg of tissue, and based on our previous experience in the recovery of proteins from the soluble plant extract, we estimate a final yield of *ca*. 4 g recombinant protein per 16 kg of fresh tissue per week (12 g per month). Just to give an idea of the potential of this production system, one ELISA plate using the format described in this study requires 0.2 mg antigen, so 12 g may yield enough antigen to fill 60,000 plates and analyze approximately 5,400,000 serum samples. According to our own calculations the approach followed in this study might reduce the production costs of serologic tests significantly, making them more affordable, especially in low-income countries. Only two purification steps were required to obtain >95% purity: A first initial affinity chromatography capture, followed by a second stage of polishing through cationic exchange. After the two processes, over 90% of the protein was recovered. The recombinant protein was identified by MALDI-TOF and proven to be recognized by a commercial antibody raised against the full viral protein. No sign of protein degradation was observed in the purification process. One of the main drawbacks of viral vectors for protein production in plants is the stability of the foreign gene in the plant cell during their replication cycle. This particular issue was addressed by IC-RT-PCR carried out on infected plants 8–12 dpi, that is, the period coinciding with tissue harvest. We found a remarkably good stability with 12.5% of the plants showing partial deletions. Under our growth conditions, plants at 12 dpi were reaching a weight of 1.2–1.8 g/leaf.

The ability of plant-made recombinant N protein to be recognized by antibodies was evaluated in 416 serum samples from infected and non-infected individuals collected during the first epidemic wave (April, 2020) affecting Spain in the context of a surveillance study focused on voluntarily participating active staff from the essential services of Madrid City Council (police, firemen, emergencies and other essential services). This panel of samples was reflecting the immunological status of this cohort of staff occupationally exposed to virus infection in a context of high virus transmission. Moreover, RT-PCR from nasal swabs was performed in each individual at the time of blood sample collection and, in some cases, also earlier, in order to verify their infection status. Thus, it was possible for a subset of serum samples used in this study to ensure they came from ongoing infections. It is widely known that there is a lag period between the detection of SARS-CoV-2 RNA in nasopharyngeal tract and the development of antibodies in serum. Thus, it has been observed that some RT-PCR positive individuals yield negative results in serologic assays (Pollán et al., 2020). This may explain that from 97 RT-PCR-positive individuals, seven had undetectable antibodies in serum by the reference ELISA and two more in the NPC-2 ELISA. Indeed, these two sera that yield different results in the two ELISAs may be due to the nature of each test: on the one hand, the reference ELISA is based on a double recognition (DR), which means that the specific antibodies in the sample are first captured by immobilized antigen, and after several washing steps, are detected by the addition of soluble HRP-conjugated antigen which binds to the free remaining antigen-binding sites in the antibodies. This type of ELISA can detect IgG, IgA, and IgM class antibodies, whereas the indirect NPC-2 ELISA used in this study was designed to detect only IgG. Therefore, it is likely that immunoglobulins other than IgG may be present in the two samples detected only in the reference test and went unnoticed in the NPC-2 ELISA. It is worth reminding here that although the indirect ELISA developed for this study was limited in this and probably other aspects, the aim of the study was to show the aptitude of the NPC-2 developed in plants as antigen valid for serological methods, rather than developing a fully functional ELISA method. Once the NPC-2 has been shown useful for detection of human IgG, it is highly likely that it would work in the same way in improved serological methods, including other ELISA formats such as double recognition (DR), competition, IgM (or IgA)-capture ELISAs. Indeed, this plant-produced antigen may also be used to develop some alternative on-site diagnostics not requiring specific equipment (multiwell plate readers), e.g., colloidal gold-based test strips, in order to make the serological diagnosis even more affordable.

**FIGURE 6:**
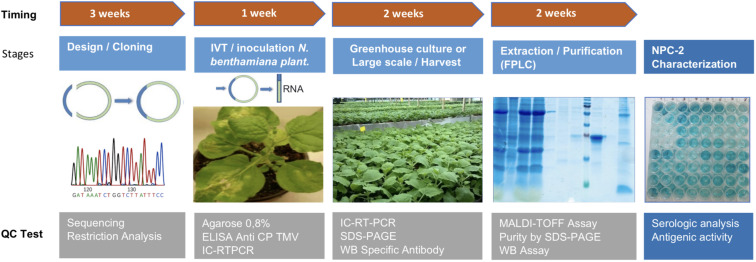
Flowchart of the complete process of production of NPC-2 from *N. benthamiana* plants. The upper timeline describes the extent of different stages: Design/Cloning (3 weeks), *in vitro* transcription and production of inoculum (1 week), plant greenhouse culture of large scale (2 weeks), Extraction/Purification of final product NPC-2 from plants (2 weeks). The lower boxes show the quality control tests associated with each stage.

Differential IgG levels could be detected by ELISA in sera from COVID-19 patients with a similar or a better sensitivity, as compared to other systems using recombinant N protein, as the reference ELISA used in this work. The assay showed a high sensitivity in patients recently infected so it can represent an interesting approach for diagnostic and epidemiological studies. The high purity, sensitivity in antibody detection, and very low level of endotoxins in the final protein preparation should make it a powerful diagnostic tool. From an industrial standpoint, the yields obtained in the purification process would allow to manufacture recombinant protein for millions of diagnostic reactions kits in 8 weeks (Figure 6).

Very recently, another study has been published showing the usefulness of other plant-produced SARS-CoV-2-derived antigens, the S (or “spike”) protein and its Receptor-Binding Domain (RBD) (Makatsa et al., 2021). Apart from the antigens used, the main difference with our study is that Makatsa et al. used a lower number of samples in the validation (77 positive, 58 negative), that IgA and IgM were also tested, and that IgA/IgG in saliva were also evaluated. Overall, plant-expressed S and RBD from SARS-CoV-2 performed well at detecting SARS-CoV-2-specific immunoglobulins, with slightly better sensitivity for S. We confirm and extend this result to N, with sensitivities, measured in RT-PCR confirmed cases, higher that those obtained with S or RBD, although the different selection of sample donors in both studies may impair a direct comparison of the results. As discussed above, serologic tests based on S (or RBD) and N are both necessary and complement each other when it comes to differentiating infection- from vaccination induced antibodies. In addition, the S protein accumulates mutations in a higher rate than in the N protein (Kaushal et al., 2020), where variable sites affect its N-terminal but not its C-terminal half, used in this study as antigen. Therefore, NCP-2-based immunoassays would be less prone to sensitivity losses due to infections by SARS-CoV2 variants.

Although not specifically tested in this study, cross-reaction with other coronaviruses currently circulating in humans (i.e., excluding SARS-1, which is not circulating anymore) is not expected due to the low percentages of identity observed in the amino acid sequence of homologous regions of our truncated version of SARS-CoV-2 nucleoprotein (NPC-2) in the human coronaviruses, such as MERS (51%), OC43 (37%), HKU1 (36%), NL63 (29%), and 229E (27%).

Upon validation of the results and technology presented in this work, we should be in a position to tackle the production of other SARS-CoV-2 proteins. This would allow increased specificity in the detection of virus variants.

Finally, it is worth to remark that in this study, technology derived from the first virus ever described (TMV, 1892) is used to produce a protein from the last human described virus (SARS-CoV2, 2019) at an industrial scale, and enables the development of cost-efficient serological tests useful to fight its infection, and INGENASA for providing the ELISA kits used as reference technique.

## Data Availability Statement

The raw data supporting the conclusions of this article will be made available by the authors, without undue reservation.

## Author Contributions

PL, LW, FP, and MJ-C conceived this article. LW, SJ, and FL designed the experiments. LW and PL drafted the first manuscript with support from FP. SJ, AR, SG, AS, IB, FL, and LW carried out the experiments. MM-C and BP-G contributed to sample management and procurement. LW and FL analyzed data and performed statistical analyses. All authors discussed the results and contributed to the final manuscript.

## Conflict of Interest

LW, SJ, AR, SG, AS, IB, and PL were employed by the company Agrenvec S.L. The remaining authors declare that the research was conducted in the absence of any commercial or financial relationships that could be construed as a potential conflict of interest.

## Publisher’s Note

All claims expressed in this article are solely those of the authors and do not necessarily represent those of their affiliated organizations, or those of the publisher, the editors and the reviewers. Any product that may be evaluated in this article, or claim that may be made by its manufacturer, is not guaranteed or endorsed by the publisher.
